# Exercise Protects against Chronic β-Adrenergic Remodeling of the Heart by Activation of Endothelial Nitric Oxide Synthase

**DOI:** 10.1371/journal.pone.0096892

**Published:** 2014-05-08

**Authors:** Liang Yang, Zhe Jia, Lei Yang, Mengmeng Zhu, Jincai Zhang, Jie Liu, Ping Wu, Wencong Tian, Jing Li, Zhi Qi, Xiangdong Tang

**Affiliations:** 1 Department of Pharmacology, Nankai University School of Medicine, Tianjin, China; 2 Departments of Histology and Embryology, Nankai University School of Medicine, Tianjin, China; Temple University, United States of America

## Abstract

Extensive data have shown that exercise training can provide cardio-protection against pathological cardiac hypertrophy. However, how long the heart can retain cardio-protective phenotype after the cessation of exercise is currently unknown. In this study, we investigated the time course of the loss of cardio-protection after cessation of exercise and the signaling molecules that are responsible for the possible sustained protection. Mice were made to run on a treadmill six times a week for 4 weeks and then rested for a period of 0, 1, 2 and 4 weeks followed by isoproterenol injection for 8 days. Morphological, echocardiographic and hemodynamic changes were measured, gene reactivation was determined by real-time PCR, and the expression and phosphorylation status of several cardio-protective signaling molecules were analyzed by Western-blot. HW/BW, HW/TL and LW/BW decreased significantly in exercise training (ER) mice. The less necrosis and lower fetal gene reactivation induced by isoproterenol injection were also found in ER mice. The echocardiographic and hemodynamic changes induced by β-adrenergic overload were also attenuated in ER mice. The protective effects can be sustained for at least 2 weeks after the cessation of the training. Western-blot analysis showed that the alterations in the phosphorylation status of endothelial nitric oxide synthase (eNOS) (increase in serine 1177 and decrease in threonine 495) continued for 2 weeks after the cessation of the training whereas increases of the phosphorylation of Akt and mTOR disappeared. Further study showed that L-NG-Nitroarginine methyl ester (L-NAME) treatment abolished the cardio-protective effects of ER. Our findings demonstrate that stimulation of eNOS in mice through exercise training provides acute and sustained cardioprotection against cardiac hypertrophy.

## Introduction

Heart failure remains a leading cause of cardiovascular morbidity and mortality, and cardiac hypertrophy is an independent and powerful predictor of heart failure [1]. Typically, cardiac hypertrophy results from pathological conditions such as hypertension, acute myocardial infarction or as a response to neurohormonal activation, often recapitulated using angiotensin receptor or β-adrenoceptor agonists [2]. It is characterized by enlargement of heart muscle, metabolic and biochemical abnormality, and reactivation of fetal cardiac genes such as atrial natriuretic factor (ANF) and β-myosin heavy chain (β-MHC) [3]. Ultimately, these lead to irreversible interstitial fibrosis, cell death and cardiac dysfunction. Thus, cardiac hypertrophy has been regarded as a potent target in preventing and treating heart failure.

Extensive data have shown that exercise training could protect against pathological cardiac hypertrophy in animal models and is associated with improved survival in humans with heart failure [4], [5]. Further studies demonstrate that phosphoinositide-3 kinase-α (PI3Kα) signaling plays a critical role in preventing pathological hypertrophy [6], [7]. Transgenic PI3Kα mice were resistant to cardiac hypertrophy and cardiac dysfunction induced by pressure overload, and their lifespan were improved [7], [8]. Over-expression of mammalian target of rapamycin (mTOR), the ‘downstream’ phosphorylation targets of the PI3Kα, was protected against cardiac dysfunction following transverse aortic constriction [9]. Interestingly, the cardio-protective effects of exercise are not confined to the period of exercise. Also, whether the PI3Kα pathway is responsible for the possible sustained protection needs to be identified.

Previous studies have shown that nitric oxide (NO) plays an important role in the modulation of cardiac hypertrophy. Augmented endothelial NO synthase (eNOS) signaling by calcium antagonist or angiotensin I converting enzyme inhibitors isassociated with improvements in myocardial remodeling and heart failure [10], [11]. Administration of the NO precursor L-arginine attenuated cardiac hypertrophy in spontaneously hypertensive rats by increasing myocardial production of NO [12]. In addition, overexpression of eNOS in cardiomyocytes was found to improve cardiac function and attenuate hypertrophy in heart failure from myocardial infarction or chronic isoproterenol infusion [13], [14]. On the other hand, there are contradictory reports showing that NO production provoked by interleukin-1β or nitroglycerin had no influence on the growth of cardiac myocytes induced by adrenergic stimulation [15] and increased NO production in the failing heart contributed to the depression of β-adrenergic responsiveness [16]. Thus, the inhibitory effects of NO on the growth of cardiac hypertrophy are controversial, and it remains unclear whether the increased level of NO signaling during exercise [17] has a part in the protective from cardiac hypertrophy.

To address these issues, we examined the duration of validity for the cardio-protective effects of exercise training against the isoproterenol-induced cardiac hypertrophy. Additionally, we investigated whether Akt/mTOR pathway contributes to the sustained cardio-protective effects of exercise. Specifically, we investigated the role that NO signaling played in mediating the cardio-protective effects of exercise.

## Materials and Methods

### Animal

C57BL/6 mice were purchased from the Military Academy of the Medical Science Laboratory Animal Center (Beijing, China). All animal were anesthetized with diethyl ether before each experiment and all efforts were made to minimize their suffering. All animal experiments were performed strictly under the guidelines on laboratory animals of Nankai University and were approved by the Institute Research Ethics Committee at the Nankai University (Permit number: 10011).

### Experimental Groups and Treatment

Mice were placed in custom-designed cages fitted with running wheels (China) for a period up to 4 weeks. Mice ran six times a week, the sessions initially lasted for 1.5 h and were increased by 15 min each day to reach 2.5h on day 5. After the exercise-training period, the running wheel was removed from the cage and the mice were allowed to rest for a 24-hour, 1-week, 2-week, or 4-week period. The control (con) and isoproterenol (ISO) groups remained sedentary during the experimental period. Isoproterenol (50 mg/kg) was injected subcutaneously (s.c.) once daily for 8 days. Mice in L-NG-Nitroarginine methyl ester (L-NAME) treatment group were administrated L-NAME (Sigma Aldrich, St. Louis, MO, USA) dissolved in drinking water at a concentration of 100 mg/L during the exercise period (ISO plus exercise plus L-NAME group) or sedentary (ISO plus L-NAME group). Water intake was measured daily by dividing the total consumption by the number of mice in each cage.

### RT-PCR and Quantitative Real-time PCR (qPCR) Assays

Total RNA in LV samples was isolated with Trizol reagent (Invitrogen, Shanghai, China). For cDNA synthesis, 1.0 µg RNA was used and reactions were carried out using reverse transcribed system (Promega, Shanghai, China). RT-PCR was performed in a Genemate thermal cycler (Jinge Instr, Hangzhou, China). Follow primers were used. For ANF 5′-GGGGGTAGGATTGACAGGAT-3′ and 5′-CTCCAGGAGGGTATTCACCA-3′; for 18s rRNA 5′-ACCGCAGCTAGGAATA ATGGA-3′ and 5′-GCCTCAGTTCCGAAAACCA-3′. for Procollagen I α I 5′-CCGCCATCAAGGTCTACTGC-3′ and 5′-GAATCCATCGGTCATGCTCT-3′; for 5′-CCCACAGCCTTCTACACCT-3′ and 5′-CCACCCAT TCCTCCCAC-3′; for fibronectin 5′-CCCACTAACCTCCAGTTTGTC-3′ and 5′-CTCTGCTGGTTCCCTTTCAC-3′. Quantitative real-time PCR was performed using SYBR Green Master Mix (Takara Bio, Inc.) as described in a Bio-Rad IQ5 detection system and the cycle threshold (CT) values were automatically determined in triplicates and averaged. All real-time PCR sample reactions were normalized to 18s rRNA expression. A standard curve was run with the dilution series of the amplified fragment allowing for mRNA copy number calculation.

### Western Blot Analysis

Frozen LV samples were homogenized and lysed in ice cold RIPA buffer (150 mM NaCl, 50 mM Tris, pH 7.5, 0.5% deoxycholic acid, 1% NP-40, 0.1% sodium dodecyl sulphate, 1 mM Na3VO4, 10 mM NaF) and a protease/phosphatase inhibitor cocktail (5872, Cell Signaling Technology, Inc, Boston, MA, USA). Protein concentration was measured using BCA protein assay Kit (Rockford, IL, USA). Samples containing 30 µg of the homogenate were resolved in 10% SDS–PAGE and transferred to polyvinylidene fluoride (PVDF) membrane (Millipore). The membrane was soaked in TBS-T/milk (5% non-fat dry milk, 10 mM Tris-HCl, pH 7.6, 150 mM NaCl and 0.1% Tween 20) for 1.5 h at room temperature and then incubated overnight at 4°C with a primary antibody. The primary antibodies to eNOS (9572), phosphorylated eNOS at both Thr1179 (9571) and Ser473 (9574), nNOs (4231), iNOs (2982), Akt (9272), phosphorylated Akt at Ser473 (9271), mTOR (2972), phosphorylated mTOR at Ser2448 (2971) were purchased Cell Signaling Technology (Boston, MA, USA). Anti-rabbit IgG with peroxidase-conjugated antibody was used as secondary antibody. Band densities were quantified using ImageJ program.

### H-E Staining

At the end of the 8-day ISO treatment period, the mice were sacrificed and the hearts were immediately removed. The left ventricles were fixed in 4% paraformaldehyde for 24 h at room temperature. The tissues were dehydrated by sequential washes with 70%, 80%, 90%, and 100% ethanol and embedded in ParaplastH X-tra Tissue Embedding Medium (McCormick Scientific). Transversal sections (5 mm) were cut starting from the base area of the left ventricle at 40-mm intervals and stained with hematoxylin and eosin for cell morphometry. The cardiomyocyte diameter was evaluated in the tissue sections using an ocular micrometer calibrated with a stage micrometer adapted to a light microscope (Olympus) at 100×magnification and analyzed using ImageJ software. Only cardiomyocytes cut longitudinally with the nuclei and cellular limits visible were used for analysis (an average of 15 cardiomyocytes for each slice). The diameter of each myocyte was measured across the region corresponding to the nucleus.

### Echocardiography

Echocardiography (Visualsonic Vevo ^2100^, 30 MHz linear signal transducer) was conducted essentially the same as described previously [18]. Briefly, averaged M-mode measures from parasternal long-axis images were recorded under isoflurane/oxygen an aesthesia after 24 h of the last injection. Inter ventricular septal (IVS) and LV posterior wall (LVPW) dimensions were taken in diastole and systole, in addition to LV internal dimensions (LVIDd and LVIDs, respectively). Fractional shortening (FS) was calculated as (LVIDd- LVIDs/LVIDd)×100 and ejection fraction (EF) as (LVIDd^3^- LVIDs^3^)/LVIDd^3^×100.

### Non-invasive (Tail-cuff) Blood Pressure and Heart Rate Measurements

Systolic blood pressure (SBP) and heart rate (HR) were measured using the tail-cuff method with a noninvasive blood pressure system (BP-98A, Softron) within 48 h after last injection. The mice were placed in a holder for three consecutive days (20 min per day) prior to beginning blood pressure measurements. On the day of blood pressure measurements, the mice were confined in small, dark holders for 15 min to 20 min prior to obtaining pressure measurements. Before and during these measurements, the holders were placed over warming pads (TMC-206, Softron). HR was monitored throughout the blood pressure measurements.

### The Isolated Heart (Langendorff) Assay

The animals (n = 4–5 mouse/group) were sacrificed 10–15 min after ip injection of 400 IU of heparin. Then the heart was carefully dissected and perfused with Krebs-Ringer solution (KRS) containing (in mmol/L): 118 NaCl, 4.7 KCl, 1.2 KH2PO4, 1.2 MgSO4, 25 NaHCO3, 11.7 glucose, 2 Na-pyruvate and 2.0 CaCl2. The perfusion fluid was maintained at 37±1°C and constant oxygenation (5% CO2/95% O2). A force transducer was attached through a balloon to the apex of the ventricles to record the LV parameters on a computer by a data acquisition system (RM6240C System, Chengdu, China). After 20–25 min of stabilization, the LV parameters, including LV systolic pressure (LVSP), LV diastolic pressure (LVDP), and maximal positive (+dP/dt) and negative (−dP/dt) time derivatives of the developed pressure were recorded for an additional 30-min period.

### NO Measurement

Plasma NO was measured by plasma nitrite plus nitrate values using a modified Griess reaction. In brief, blood was sampled from the aorta and was centrifuged at 5000 rpm for 10 min at 4°C. The supernatants were extracted three times. A total of 50 µL of the samples were incubated with 50 µL of the Griess reagent (part I: 1% sulphanilamide; part II: 0.1% naphthylethylene diamide dihydrochloride and 2% phosphoric acid) at room temperature. Ten minutes later, the absorbance was measured at 540 nm using an automatic plate reader, and the NOx concentrations were expressed as mmol·L-1 and calculated using a standard curve of NOx from commercially available kits (Beyotime Institution of Biotechnology, China).”

### Statistical Analysis

Results are expressed as means ± SEM. One-way ANOVA followed by LSD test or Student’s t test was performed as implemented in SPSS as described. A value of *p*<0.05 was accepted as statistically significant.

## Results

### 1. Exercise Training Inhibits Physical Changes Following Chronic Isoproterenol Treatment

**Figure 1:**
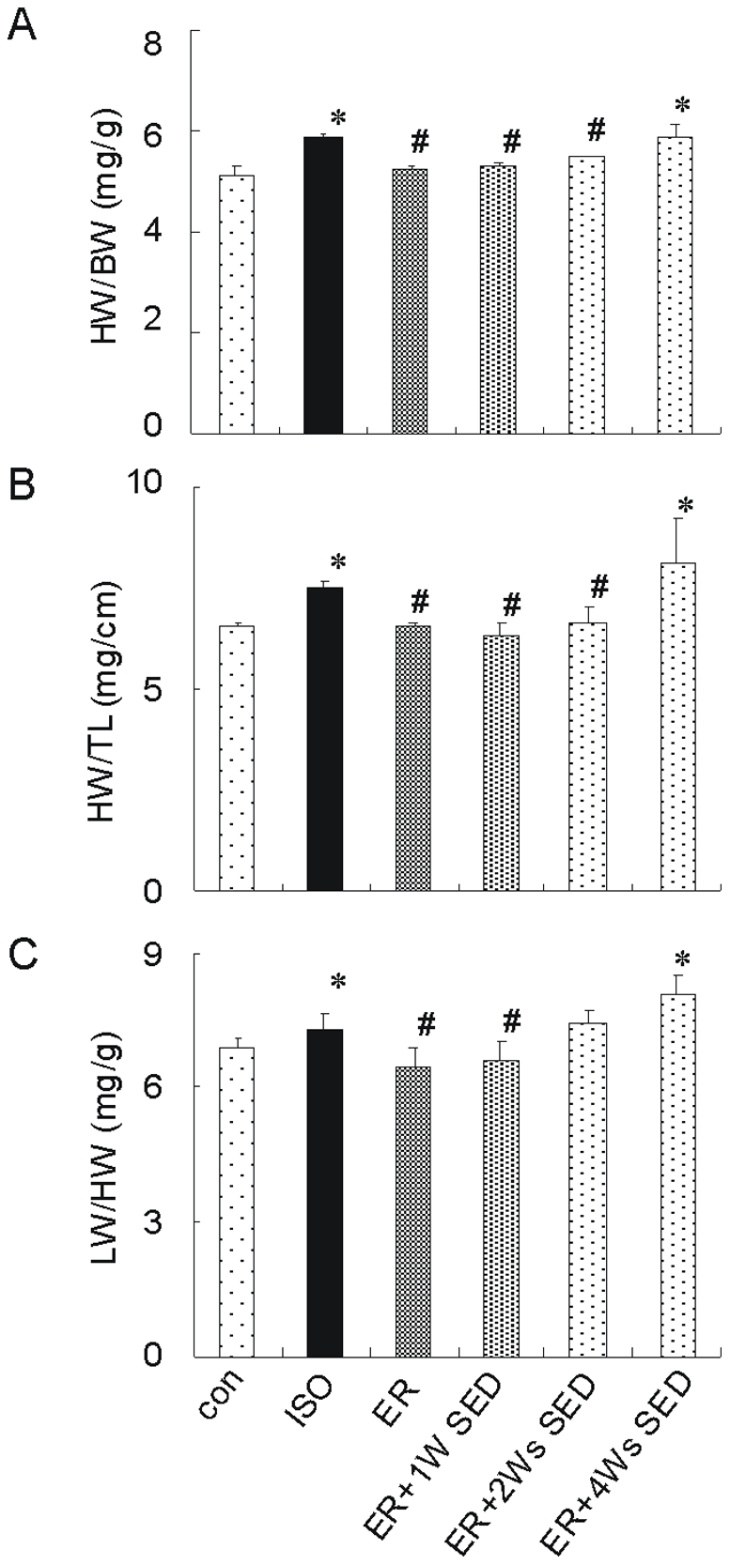
Exercise training reduced the myocardial hypertrophy induced by isoproterenol. Mice were housed in cages fitted with running wheels and allowed to exercise training for 4 weeks. 50/kg isoproterenol was injected intraperitoneal for 8 days either immediately after the training period (ER, n = 7) or 1 week (ER +1W SED, n = 7) or 2 weeks (ER +2Ws SED, n = 7) or 4 week after the training period (ER +4Ws SED, n = 7). Isoproterenol mice (ISO, n = 7) or control mice (con, n = 7) were housed in cages without running for the same durations as were the ER mice and then injected with ISO or vehicle control. (A) heart weight/body weight (HW/BW) ratio, (B) HW/tibia length (TL) ratio and (C) Lung weight (LW)/BW ratio for all groups. Values are means ± SEM. * *p*<0.05 versus con group, ^#^ *p*<0.05 versus ISO group.

To determine whether exercise training can attenuate the morphological changes induced by isoproterenol injection, mice were made to run on a treadmill six times a week for 4 weeks and isoproterenol was administered for 8 days after training (ER mice). ISO mice or con mice were housed in cages without running for the same durations as were the ER mice and then injected with isoproterenol or vehicle control. As shown in Figure 1, chronic isoproterenol treatment resulted in a significant increase in normalized HW in ISO mice in comparison to con mice [HW/body weight (BW) 5.8±0.1 vs. 4.6±0.1, HW/tibia length (TL) 7.5±0.3 vs. 6.1±0.3, respectively, n = 10, *p* < 0.05], consistent with increase in lung weight (LW)/BW [7.3±0.3 vs. 5.9±0.6, n = 10, *p* < 0.05]. As expect, the ER mice displayed a 10% reduction in HW/BW (5.2±0.2 vs. 5.8±0.1, n = 10, *p* < 0.01) and a 12% reduction in HW/TL (6.5±0.4 vs. 7.5±0.3, n = 10, *p* < 0.01). LW/BW in ER mice was also decreased obviously compared to ISO group (6.5±0.3 vs. 7.3±0.3, n = 10, *p<*0.05).

Then, we evaluated how long the cardio-protective effects of ER could be maintained after the mice stopped training. For these experiments, mice were allowed to exercise for 4 weeks and then were removed from the cages for a period of either 1 week (ER +1W SED) or 2 weeks (ER +2Ws SED) or 4 weeks (ER +2Ws SED). In the end of the cessation, isoproterenol was injected intraperitoneal for another 8 days. Interestingly, the ER mice with 1 week cessation also displayed about 9% reduction (*p* < 0.01 versus SED; Figure 1B) in normalized HW [HW/BW: 5.3±0.1 vs. 5.8±0.1; HW/TL: 6.3±0.3 vs. 7.5±0.3, n = 7] and a 9% reduction in LW/BW (6.7±0.6 vs. 7.3±0.3, n = 7) compared to ISO mice. The ER +2Ws SED mice also showed a significant decrease in normalized HW compared to ISO mice. However, there were no differences between ER +4Ws SED and ISO groups in HW/BW, HW/TL and LW/BW (5.8±0.2 vs. 5.8±0.1, 8.1±1.0 vs. 7.5±0.3 and 8.0±0.4 vs. 7.3±0.3, respectively, n = 7, NS). These data demonstrated that the cardio-protection of ER against cardiac hypertrophy disappeared following 4 weeks cessation of the training.

### 2. Exercise Training Attenuates the Echocardiographic and Hemodynamic Changes after β-adrenergic Overload

**Figure 2:**
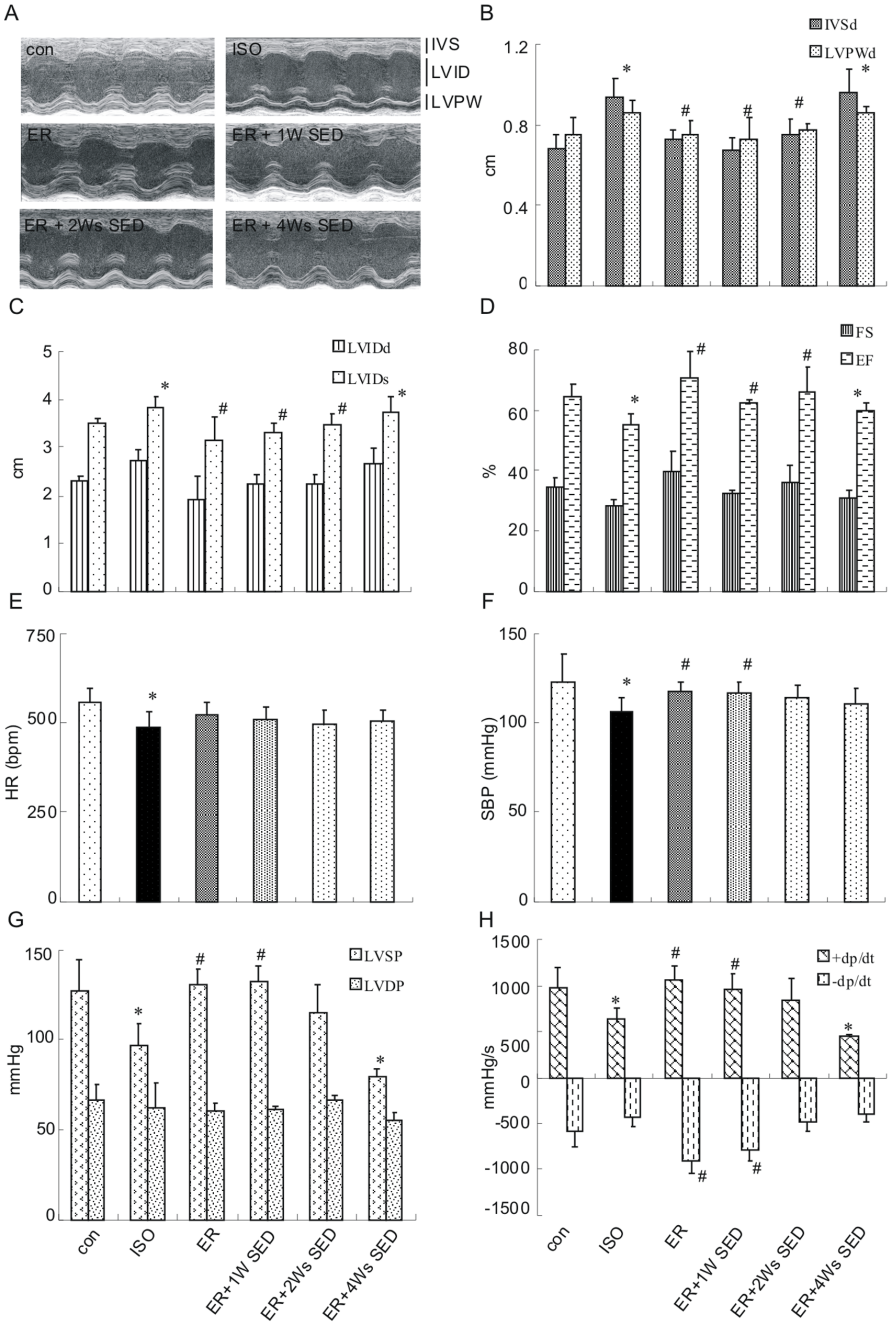
Exercise training attenuates the echocardiographic and hemodynamic changes induced by isoproterenol injection. Echocardiography and hemodynamics were performed within 48-papillary level. (A) Representative long-axis parasternal echocardiographic images, IVS, interventricular septum; LVID, left ventricular internal dimension; LVPW, left ventricular posterior wall. (B, C) IVSd and LVPWd represent end-diastole IVS and LVPW measurement, LVIDd/s represent end-diastole and end-systole LVID measurements, respectively. (D) FS = [(LVIDd - LVIDs/LVIDd)×100] and EF = [(LVIDd3- LVIDs3)/LVIDd3×100]. (E) HR, heart rate. (F) SBP, systolic blood pressure, (G)LVSP, LV systolic pressure; LVDP, LV diastolic pressure and (H) ±dP/dt, maximal positive (+dP/dt) and negative (−dP/dt) time derivatives of the developed pressure are shown. Values are means ± SEM. * *p*<0.05 versus con group, ^#^ *p*<0.05 versus ISO group.

To examine the potential remodeling after the stress stimulus, echocardiography and hemodynamics were performed after the last injection. On transthoracic echocardiography, isoproterenol treatment induced significant increases in IVS and LVPW thickness compared with control group (0.93±0.08 vs. 0.68±0.07 and 0.85±0.06 vs. 0.75±0.08 mm, respectively, n = 7, *p*<0.05, Figure 2B). This was associated with ventricular dilatation (3.84±0.26 vs. 3.52±0.11 and 2.73±0.23 vs. 1.92±0.06 mm, respectively, for LVIDd and LVIDs, n = 6, *p*<0.05, Figure 2C) and impairment of cardiac function (FS: 28±2 vs. 34±3%; EF: 55±3 vs. 64±4%, n = 6, *p*<0.05, Figure 2D). Conversely, compared with isoproterenol treatment group, there was apparent reduction of IVS and LVPW thickness in the ER group (0.73±0.04 vs. 0.93±0.08 mm; 0.66±0.07 vs. 0.85±0.06 mm, respectively, n = 7, *p*<0.05), consistent with attenuation of contractile impairment (FS: 39±7 vs. 28±2%; EF: 70±9 vs. 55±3%, n = 7, *p*<0.05). Furthermore, the ER +1W SED and ER +2Ws SED mice also displayed a significant reduction in mean LV wall thickness and internal LV dimensions compared to ISO mice (*p<*0.01 versus ISO; Figure 2). Likewise, FS and EF demonstrated obviously improvement compared to ISO mice. Conversely, all changes in these measures were significantly deteriorated in ER +4Ws SED mice compared to ER mice or con mice.

On hemodynamics measurements, HR in ISO group was lower than that in control group (485±45 vs. 558±40 mmHg, n = 7; *p*<0.05, Figure 2E), and SBP were decreased in ISO group (106±7 vs. 122±15 mmHg, n = 7; *p*<0.05, Figure 2F). Also, the LVSP and +dP/dt were significantly lower in the ISO group compared with control group (*p*<0.05, Figure 2G, 2H). In contrast, there was apparent increase in SBP, LVSP and +dP/dt in ER group compared to ISO group, while there is no difference in HR or LVDP between these two groups. The significant increases in LVSP and +dP/dt were also found in ER +1W SED mice and ER +2W SED mice. However, there was no difference in those parameters between ER +4Ws SED and ISO group. These results indicated that exercise-induced preservation from cardiac hypertrophy disappeared at 4 weeks after the cessation of the training.

### 3. ER Training Attenuates Fibrosis and Fetal Gene Reactivation Induced by ISO Injection

**Figure 3:**
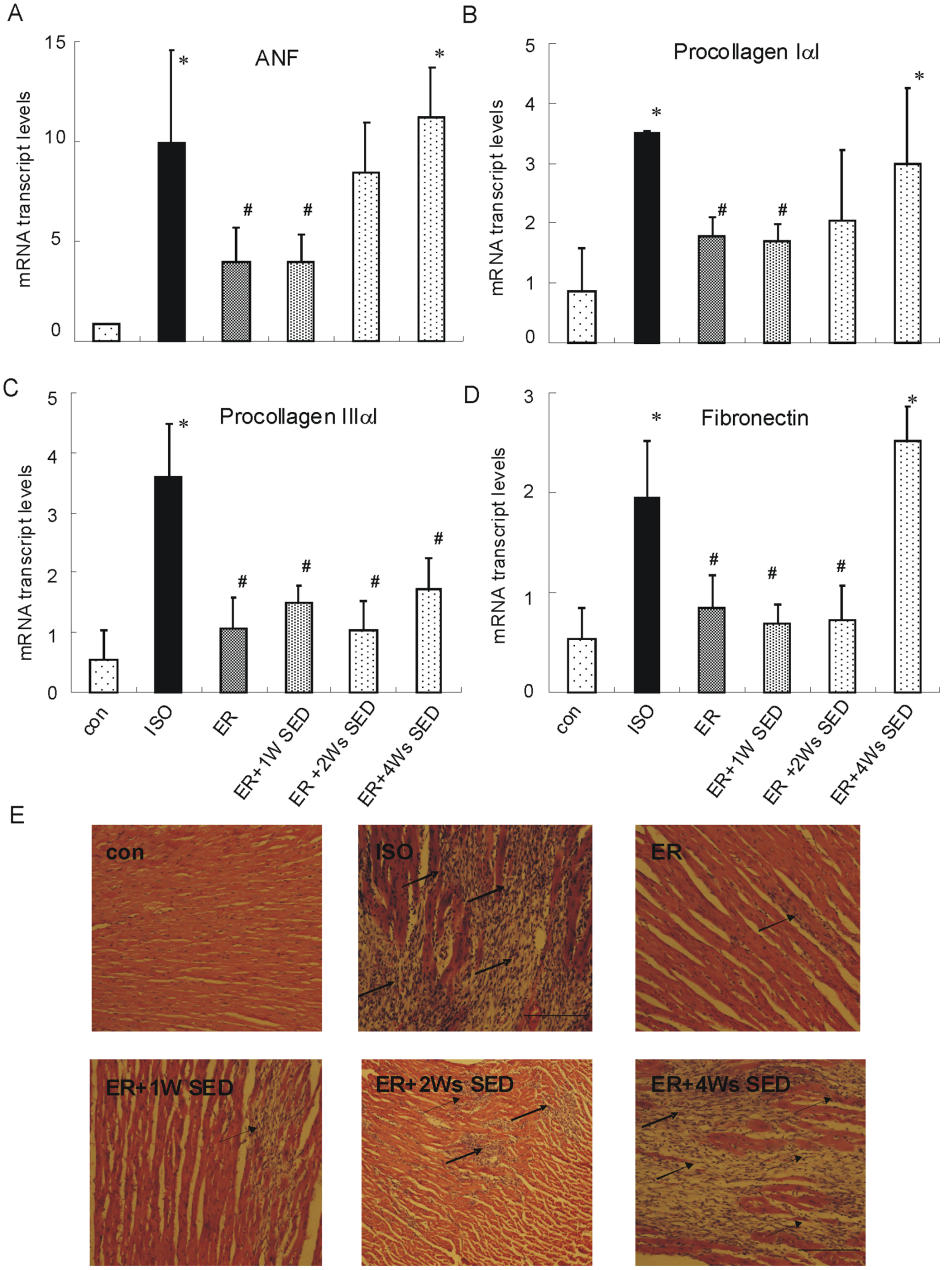
ER training attenuates fibrosis and fetal gene reactivation induced by isoproterenol injection. (A–D), Quantification of the mRNA transcript abundance for ANF, procollagens IaI, IIIaI, and fibronectin, n = 5/group, All transcript results are normalized to 18-s mRNA levels. * *p*<0.05 versus con group, ^#^ *p*<0.05 versus ISO group. (E), Histological sections of hearts by H-E staining. The sections were photographed under 100-fold microscopy.

Then, to examine whether the gross morphological changes in response to stress were mirrored at the cellular level, myocardial fibrosis and the evidence of gene re-activation were measured by HE staining and real-time PCR. As shown in Figure 3, isoproterenol treatment induced ANF expression by ∼ 12-fold. Also, there were significant increases in genes encoding procollagen IaI, IIIaI, and fibronectin, consistent with large area of interstitial fibrosis by histology. In ER and ER +1W SED mice, compared to ISO mice, the expression of ANF was significantly decreases to ∼ 5-fold and the expression of procollagen IaI, IIIaI, and fibronectin were also down-regulated obviously, as well as less area of interstitial fibrosis. In contrast, mice in ER +2Ws SED and ER +4Ws SED group displayed no different changes in ANF expression compared to ISO mice. Large area interstitial fibrosis was also found in ER +4Ws SED mice by HE staining. All the results confirmed that the cardio-protective effects of exercise were lost following 4 weeks cessation of the training.

### 4. The Effect of ER Training on the Phosphorylation Status of Akt and mTOR

**Figure 4:**
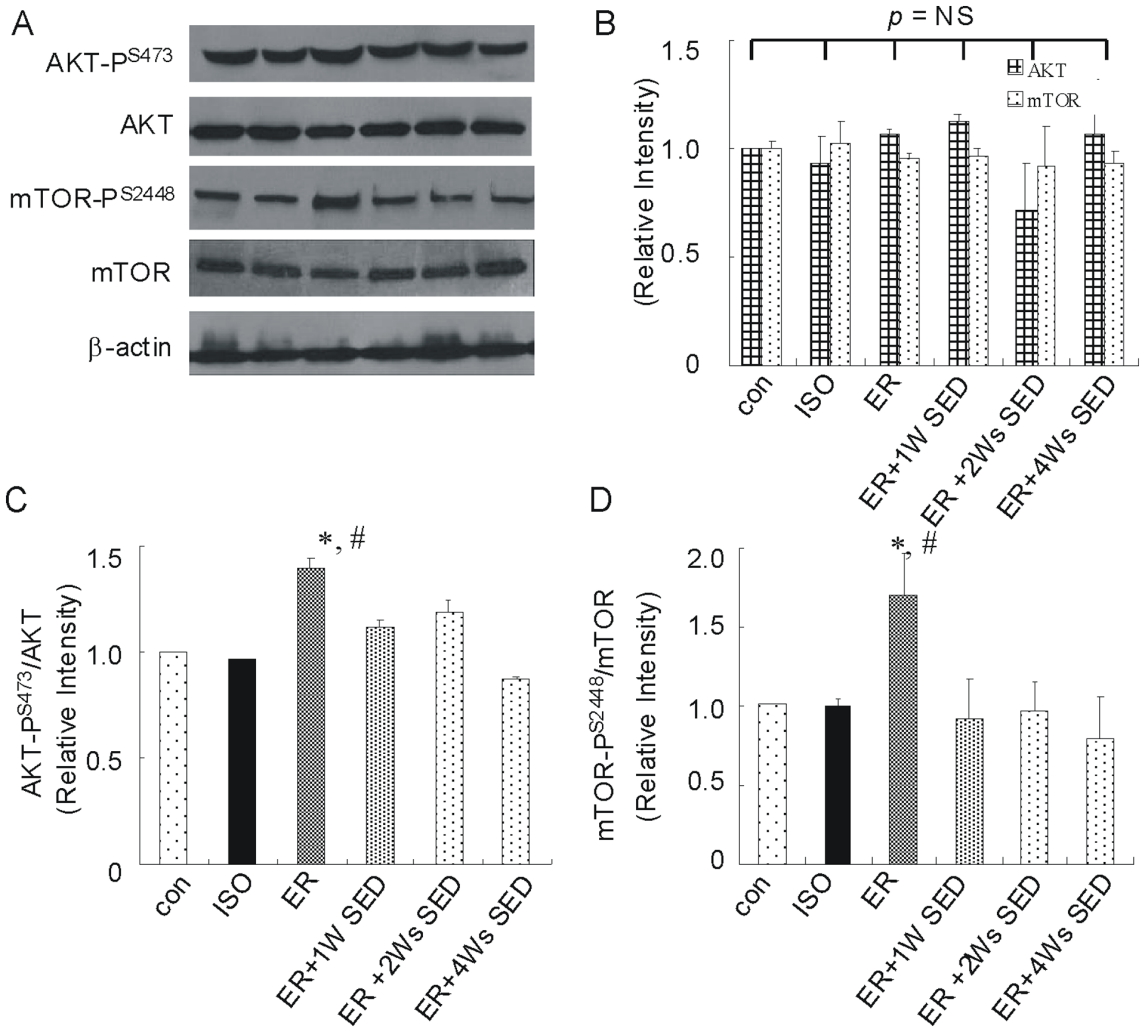
ER training altered the phosphorylation status of AKT and mTOR in comparison with control mice. (A) Representative immunoblots and densitometric analysis of total AKT and mTOR (B) and phosphorylated AKT at serine 473 (AKT-P^S473^) (C) and phosphorylated mTOR at Ser 2448 (mTOR-P^S2448^) (D) following 4 weeks of exercise training. Values are means ± SEM. * *p*<0.05 versus con group, ^#^ *p*<0.05 versus ISO group.

To assess the mechanism by which ER inhibits isoproterenol-induced cardiac hypertrophy, we examined the expression and phosphorylation status of several cardio-protective signaling molecules, which purported to play a role in mediating exercise-induced cardio-protection. For these experiments, phosphorylation site-specific antibodies were used to probe immunoblots prepared from the heart tissue. As shown in Figure 4, the level of serine 473 phosphorylation of Akt in ISO-treated hearts was not reduced significantly. In contrast, and as reported previously [19], a significantly increase of the phosphorylation of Akt was observed in the hearts of ER group. It has been shown that mTOR is the ‘downstream’ phosphorylation targets of the kinase Akt [20]. As with Akt, mTOR was also obviously phosphorylated in the hearts of ER mice (Figure 4D). However, the phosphorylation status of all those molecules did not increase obviously in the hearts of the ER +1W SED or ER +2Ws SED group. Additionally, total protein expression levels of Akt or mTOR did not change obviously in either ER or isoproterenol-treated groups. All the results suggested that some other cardio-protective molecules are responsible for this sustained protection.

### 5. ER Training Alters the Phosphorylation Status of Endothelial Nitric Oxide Synthase and NO Metabolite

**Figure 5:**
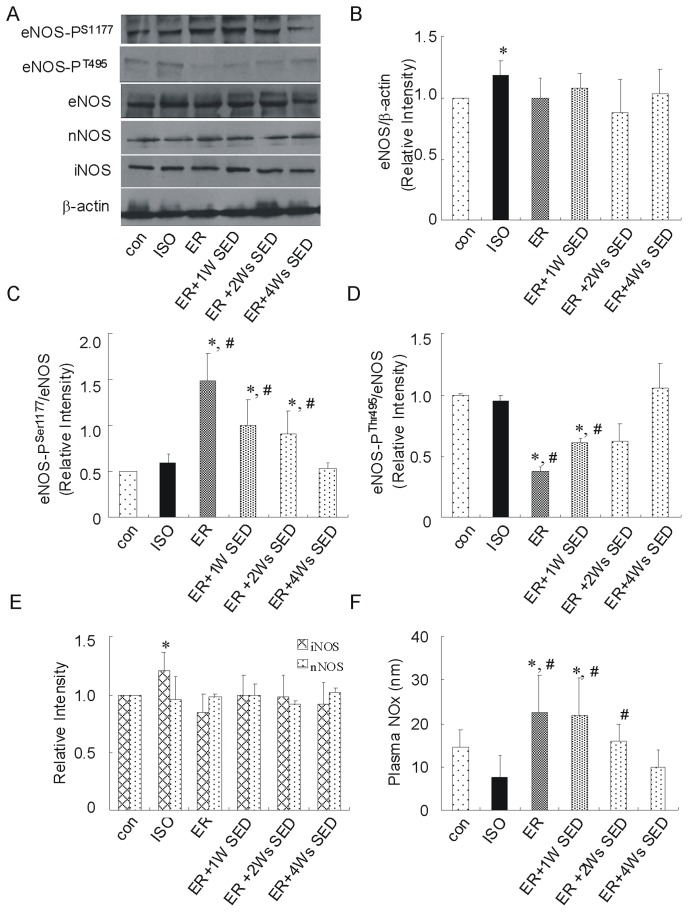
ER training altered the phosphorylation status of cardiac eNOS and NOx production. (A) Representative immunoblots and densitometric analysis of total eNOS (B) phosphorylated eNOS at serine residue 1177 (eNOS-P^S1177^) (C) phosphorylated eNOS at threonine residue 495 (eNOS-P^T495^) (D) iNOS and nNOS (E) following 4 weeks of exercise training. (F) The NOx production was quantified by measurements of nitrite and nitrate in mouse plasma. Values are means ± SEM. * *p*<0.05 versus con group, ^#^ *p*<0.05 versus ISO group.

There are there isoforms of neuronal, inducible, and endothelial nitric oxide synthase (NOS) in the heart [21] and we examined the effects of ER on NOS expression and phosphorylation status by western-blot. As shown in Figure 5, significant increases in total eNOS were observed in the hearts of the ISO group but not in the other groups. However, the phosphorylation of eNOS did not change obviously in the ISO group. In contrast, ER training promoted a significant increase in the phosphorylation of eNOS at serine residue 1177 (eNOS-P^S1177^; phosphorylation here increases enzyme activity) and the dephosphorylation at threonine residue 495 (eNOS-P^T495^; phosphorylation here inhibits the enzyme activity), whereas the total eNOS did not change significantly. Importantly, the alterations in the phosphorylation status of eNOS were still present in 2 weeks after the 4-week ER training (especially eNOS-P^S1177^, p<0.05 versus con). Additionally, there was an obvious increase in iNOS expression in the ISO group compared to con group. The expression of nNOS remained unchanged in response to ER training or ISO injection (Figure 5E). All the results indicated that the activation of eNOS contributed to the sustained cardio-protection against cardiac hypertrophy by exercise training.

Next, we evaluated the effects of exercise training on the levels of total nitric oxide in the plasma. As shown in Figure 5F, a significant increase in the levels of nitric oxide was observed in the plasma of the ER group compared to ISO group. Importantly, these elevations were still present 2 weeks after the training. In contrast, the ER +4 week SED mice were not protected against cardiac hypertrophy, whereas no changes in plasma NO metabolite levels were found in this group compared to ISO treated group, suggesting that the sustained cardio-protective effects of exercise are lost when NO metabolite levels are not elevated.

### 6. The Cardio-protective Effects of TR Vanish by L-NAME Treatment

**Figure 6:**
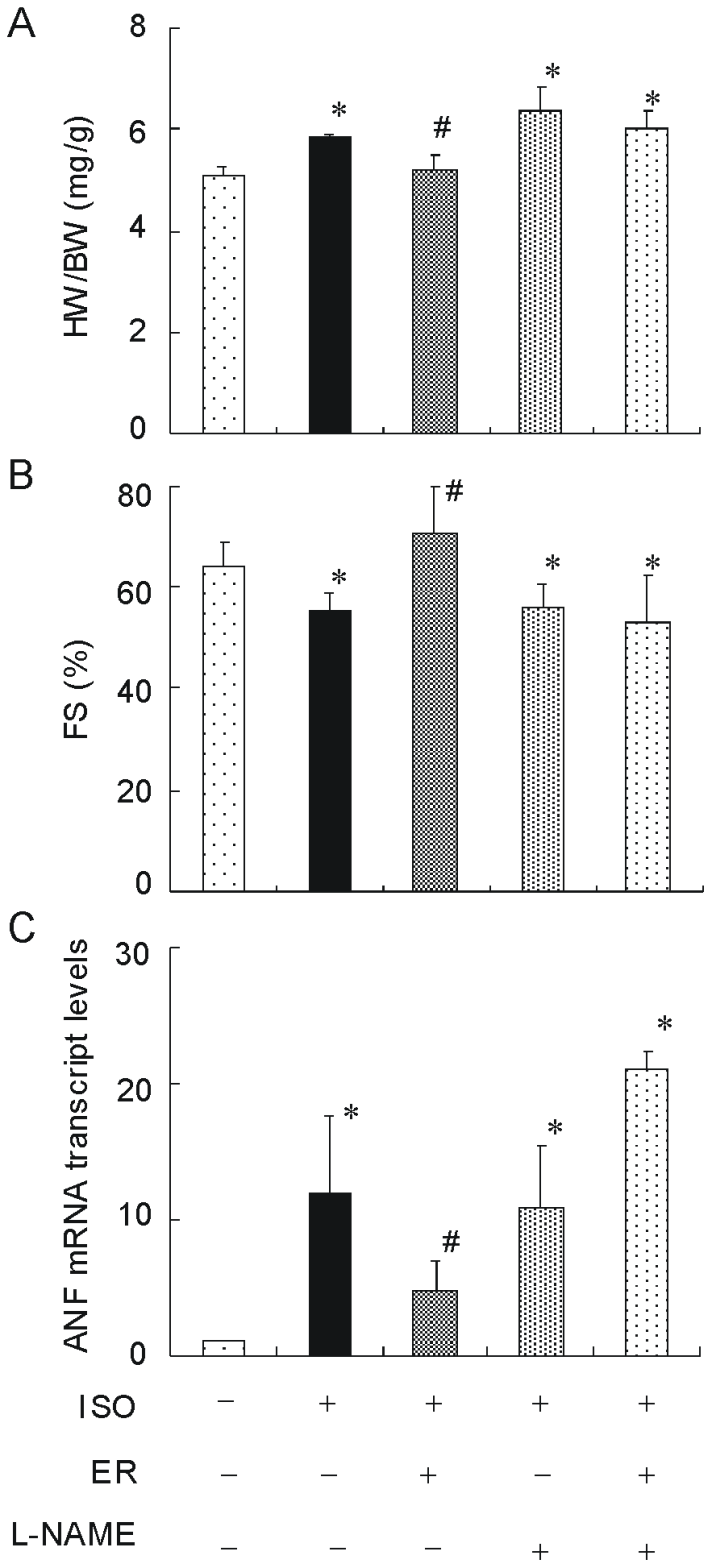
L-NAME abolishes the cardio-protective effects of ER. (A) HW/BW (B) FS and (C) ANF expression were determined following 4 weeks of exercise training and/or isoproterenol injection and/or L-NAME administration. Values are means ± SEM. * *p*<0.05 versus con group, ^#^ *p*<0.05 versus ISO group.

Additionally, to investigate whether NOS pathway was critical for the cardio-protection afforded by ER training, a nonselective NOS inhibitor, L-NAME was used to abolish the effect of ER on NOS pathway. As shown in Figure 6, exercise training significantly prevented isoproterenol-induced changes in HW/BW, FS and ANF expression. However, this preventive effect was significantly abolished by co-administration with L-NAME. In contrast, L-NAME alone did not further increase cardiac hypertrophy in ISO treated mice. These data demonstrated that the cardio-protection effect of ER against cardiac hypertrophy disappeared after NOS inhibition.

## Discussion

The present study showed that exercise-induced cardioprotection against ISO induced cardiac hypertrophy lasted for at least 1 week after cessation of exercise training. Importantly, we observed that cardio-protection persists in the absence of elevated myocardial levels of PI3K-Akt-mTOR pathway at 1 week post-exercise. Further study showed that the elevation of the phosphorylation status of eNOS plays a role in mediating the acute and sustained cardio-protective effects of exercise.

Our results confirm previous data that short-term endurance exercise training results in cardioprotection against cardiac hypertrophy. In addition, our data show that acute exercise training is associated with double fold increases in myocardial levels of Akt and mTOR, which are widely reported in the literature [7]. Our studies further confirmed that the elevation of PI3K-Akt pathway disappeared after 1 week cessation of exercise. It is generally accepted that PI3K/Akt pathway signaling are activated during exercise, so it is critical for physiological exercise-induced growth of the heart but not pathological hypertrophy [4]. The duration of this effect has not been reported. Eunhee Chung *et al* reported that phosphorylation of Akt/mTOR was maximal at mid-(11 day gestation) but not the late (18–19 days gestation) stage of pregnancy [22]. All these results support the previous findings that signaling molecules are activated rapidly and precede the increase in heart weight in response to exercise. On the other hand, it has been shown that exercise training associated with activation and pressure overload after TAC partially inactivates the Akt/mTOR pathway and downstream substrates of the hearts [19]. In my study, the ISO injection may have a part in the inactivation of the phosphorylation of Akt/mTOR.

Modulation of cardiac function by NO is complex and multifaceted. Experimental studies indicated that a decrease in NO bioavailability is associated with heart failure and actually exerts deleterious effects during heart failure [23], [24]. Also, NO plays a critical role in mediating the protective effects associated with exercise. It has been shown that increased vascular wall shear stress induced by exercise increases the expression and activity of vascular eNOS, which subsequently increases the production and bioavailability of NO throughout the body [25]. Further studies confirmed that exercise increased the expression of eNOS-P^Ser1177^ and decreased the expression of eNOS-P^Thr495^ without altering the expression of total eNOS and protected the heart from myocardial ischemia-reperfusion (I/R) injury [17]. In the current study, we confirmed this finding and demonstrated that the elevation of the phosphorylation status of eNOS attenuated the cardiac hypertrophy induced by chronic ISO injection. We found that the cardioprotective effects of exercise disappeared at 4 weeks after the cessation of the exercise training or by L-NAME injection. All these findings support the idea that the activation of NO exerts beneficial effects in the setting of cardiac hypertrophy and heart failure.

It has been shown that endothelial-myocardial interactions are important in maintaining physiological regulation of cardiomyocyte [26]. ENOS is constitutively expressed in endothelium [27], and eNOS-derived NO serves as a critical endothelium-derived modulator that maintains normal function of the vasculature [28]. Recently, Thorsten et al reported that addition of endothelial to cardiomyocyte significantly increased NO production and protected cardiomyocyte from I/R injury, and endothelial dysfunction by triton X-100 incubation attenuated the recovery of cardiac function during reperfusion in Langendorff-perfused hearts subjected to I/R injury [29]. The present study showed that the increased eNOS phosphorylation and NO production contributed to the sustained cardio-protection against cardiac hypertrophy by exercise training. However, whether these elevations are localized at myocardial and/or endocardial needs to be clarified in further studies.

On the other hand, however, it has shown that NO contributes to the pathogenesis of heart failure [30]. The current study does not support the idea of NO as a deleterious mediator of heart failure. Although not the focus of our study, it seems that overproduction of NO via iNOS actually causes negative effects during heart failure due to the high capacity of iNOS to produce NO [31]. In our study, as reported previously [32], a significant increase in the expression of iNOS was also noted in ISO-treated mice. It has been confirmed that different NO synthase isoforms allows NO signals to have independent, and even opposite, effects on cardiac phenotype [33]. As such, more studies of iNOS and eNOS in heart failure are required to fully elucidate this complex physiological system.

It has been shown that the activation of eNOS during exercise can be caused by shear stress, inducing a signaling cascade involving Akt, PKA, or AMPK [34]–[36]. Recently, Zhang et al reported that inhibition of Akt signaling with wortmannin blunts the increase in the expression of eNOS-P^Ser1177^ in mice subjected to treadmill running without altering the expression of phosphorylated AMPK [37]. However, since wortmannin did not completely attenuate the increase in the expression of eNOS-P^Ser1177^, these data suggest that other signaling molecules may regulate the expression of eNOS-P^Ser1177^ during exercise [37]. Further studies showed that β_3_-ARs were involved in this process whereas the increase in the expression of eNOS-P^Ser1177^ was blunted in the hearts of β_3_-AR-deficient mice [17]. Recent reports showed that calcium dependent pathway have a key role in the modulation of cardiac myocyte eNOS activation [38]. In the current study, a significant increase of the phosphorylation of Akt/mTOR was observed in the hearts of ER group but not in the ER +1W SED or ER +2Ws SED group. In contrast, the alterations in the phosphorylation status of eNOS were still present at 2 week after the end of the 4-week ER training period, and it seems other signaling molecules regulate the activation of eNOS during exercise.

In conclusion, the current study demonstrates that 4 weeks of exercise training provide acute and sustained cardioprotection against cardiac hypertrophy induced by ISO injection. Further study shows that the effect of protection remains for at least 2 weeks after cessation of exercise training. Importantly, we observed that the increase of eNOs signaling molecules contributed to protect the heart against hypertrophy. In the future, therapies might be developed to improving vascular eNOS function as a means to improve clinical outcomes in patients with congestive heart failure.
